# Could the Suboccipital Release Technique Result in a Generalized Relaxation and Self-Perceived Improvement? A Repeated Measure Study Design

**DOI:** 10.3390/jcm13195898

**Published:** 2024-10-02

**Authors:** Rob Sillevis, Anne Weller Hansen

**Affiliations:** Department of Rehabilitation Sciences, Florida Gulf Coast University, 10501 FGCU Boulevard South, Marieb 435, Fort Myers, FL 33965, USA; awhansen@fgcu.edu

**Keywords:** nuchal line inhibition, suboccipital release technique, manual therapy, brainwave (EEG)

## Abstract

**Background:** Musculoskeletal disorders such as cervicogenic headaches present with suboccipital muscle hypertonicity and trigger points. One manual therapy intervention commonly used to target the suboccipital muscles is the suboccipital release technique, previously related to positive systemic effects. Therefore, this study aimed to determine the immediate and short-term effects of the Suboccipital Release Technique (SRT) on brainwave activity in a subgroup of healthy individuals. **Methods:** Data were collected from 37 subjects (20 females and 17 males, with a mean age of 24.5). While supine, the subjects underwent a head hold followed by suboccipital release. A total of four 15 s electroencephalogram (EEG) measurements were taken and a Global Rating of Change Scale was used to assess self-perception. **Results:** There was a statistically significant difference (*p* < 0.005) in various band waves under the following electrodes: AF3, F7, F3, FC5, T7, P7, O1, O2, P8, T8, and FC6. An 8-point range in the Global Rating of Change Scores with a mean score of 1.649 (SD = 1.719 and SE = 0.283) supported the hypothesis of a self-perceived benefit from the intervention. **Conclusions:** The results of this study indicate that the suboccipital release technique significantly affects brain wave activity throughout different brain regions. This change is likely not the result of any placebo effect and correlates highly with the subject’s self-perception of a change following the intervention. These findings support the clinical use of the suboccipital release technique when a centralized effect is desired.

## 1. Introduction

One of Western society’s most common postural defaults is forward head posture [1,2]. In this posture, the head is misaligned with the trunk, resulting in lower and mid-neck flexion, suboccipital extension, and the anterior placement of the head relative to the body axis. This position results in the adaptation of the musculoskeletal system to maintain this misaligned head position. Typically, adaptations include the shortening and tightening of the posterior suboccipital muscles, which can lead to symptomatic dysfunctions in the upper cervical spine [3,4]. The prevalence of neck pain related to forward head posture ranges between 50 and 64% in Western Society [5,6].

Healthcare providers often use manual therapy techniques to manage upper cervical spine disorders [1,7,8]. Such disorders include cervicogenic headaches, trigger points, and muscle hypertonicity. The suboccipital muscles are amongst the muscles in the body most affected by trigger points [9]. Fernandez et al. [10] identified trigger points in the suboccipital muscles of patients with forward head posture in both symptomatic and asymptomatic individuals. One manual therapy intervention commonly used to target the suboccipital muscles directly is the suboccipital release technique (SRT), also called nuchal line inhibition [11].

Previous research has demonstrated the positive short-term effect of the SRT on suboccipital muscle tone and pain in subjects with neck pain [12,13,14]. There is conflicting evidence regarding the more generalized effect of the SRT on the central nervous system. An immediate change in the hamstring length after performing the suboccipital release technique was shown by Cho et al. [15]. This generalized central effect of the SRT was supported by Metzler-Wilson et al. [16], who demonstrated an immediate change in the functioning of the autonomic nervous system. However, Sillevis et al. [11] was not able to demonstrate any autonomic changes following the SRT using an automated pupillometry method, despite the fact that the subjects in that study reported a positive change on the global rating of change scale. Additionally, subsequent to the neurogenic effects of the SRT, the direct anatomical relationship between the suboccipital muscles and the central nervous system should be considered when determining the possible effects of the SRT. Scali et al. [17] and Pontel et al. [18] identified facial tissues connecting the rectus capitis posterior major and minor and the obliquus capitis inferior with the posterior aspect of the upper cervical dura through the C1-C2 interspace. Furthermore, collagenous connections between the nuchal ligament and the dura have been identified [19]. The significance of these anatomical connections to the dura is that suboccipital muscular contractile actions and tonicity directly impact the dura, which might impact the central nervous system.

One way to evaluate changes in the central nervous system is measuring brainwave activity. The electroencephalogram (EEG) is an efficient tool for gathering real-time data on brainwave activity [20]. Brainwaves represent the fluctuations in the voltage of current passing through brain neurons and are dependent on the activity of the brain [21]. Five different brainwave types have been identified in the human brain [22]. Gamma waves are correlated with sensory processing, concentration, and problem-solving [23]. Beta waves are linked with active thinking and focus processes, such as decision-making and talking [21]. Alpha waves occur mostly during activities such as daydreaming or meditation, and Theta waves represent drowsiness [24]. Lastly, Delta waves are correlated with sleep [25]. If the SRT truly has a centralized effect on the nervous system, a change in EEG activity in different parts of the brain should be measurable. Therefore, this study aimed to further examine any immediate and short-term effects of the SRT on the central nervous system by measuring its effect on brainwave activity in a subgroup of healthy individuals. It was hypothesized that reducing muscle tone should change the brainwave activity related to their experience.

## 2. Material and Methods

### 2.1. Subjects

This repeated measures study design used a method of convenience sampling with a within-subject repeated measures design. A power analysis using G*power (Dusseldorf, Germany), version 3.1, was performed a priori, assuming a normal distribution of data in combination with a power of 0.80, alpha of 0.5, a medium effect size of 0.50, and three measurements. The G*power 3.1 *t*-test, with a mean difference from the constant, indicated that the minimum number of required subjects was 34.

#### Inclusion Criteria

To participate in this study, all subjects had to be between the ages of 18 and 65 and be able to read English so that proper written consent could be given. All available subjects were screened using the eligibility criteria by a licensed physical therapist to find any potential reasons that they could not undergo the testing. The exclusion criteria were a history of upper cervical surgery, current chiropractic and or physical therapy treatment, a positive history of concussion, a traumatic brain injury, or any history of brain damage due to the potential for atypical neural signaling because of neuroplastic changes after the injury. This study received institutional review board approval from Florida Gulf Coast University (IRB# 202064).

### 2.2. Study Protocol

#### 2.2.1. Automated Measures

**Figure 1:**
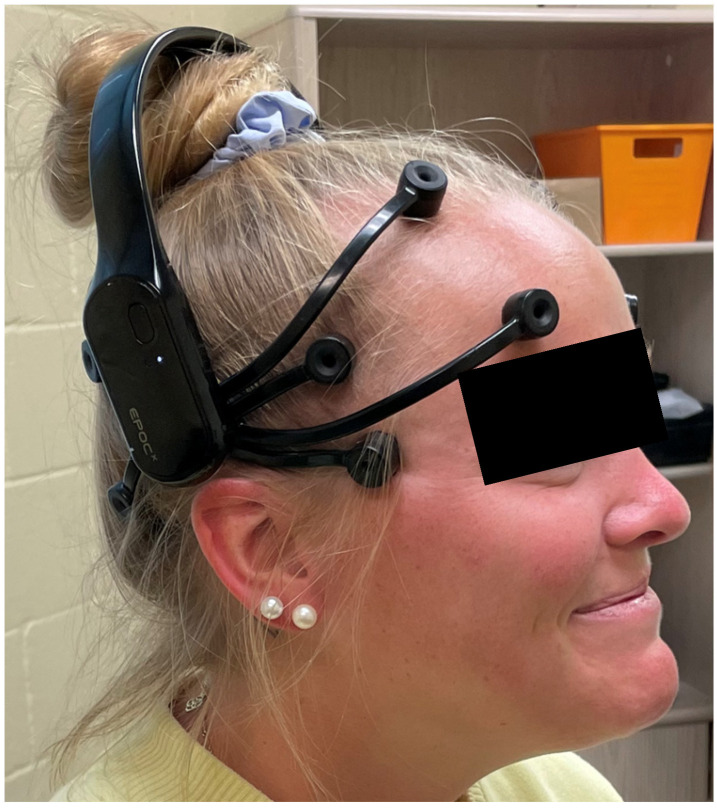
EMOTIV EPOC+ frontal, parietal, and temporal electrode placement on the head.

All testing was performed in the same room to minimize the effect of any external electrical interference on the EEG data collection process. The Emotiv + EPOC EEG device (San Francisco, CA, USA) was turned on 30 min before data collection to allow the machine to warm up. Each participant entered the testing room after providing consent. Subjects were seated to apply the Emotive EPOC+ headset by Researcher I (Figure 1).

The Emotiv EPOC+ headset is connected to the processing Emotiv Pro software (https://www.emotiv.com/products/emotivpro accessed on 1 September 2024) by Bluetooth. The EPOC+ collects brainwave data with a frequency of 128 HZ concurrently in 14 active electrodes and measures five different types of brainwaves: Theta waves are measured between the 8 and 13 Hz frequency band, Alpha waves are measured between the 8 and 13 Hz frequency band, Beta low waves are measured between the 13 and 15 Hz frequency band, Beta high waves are measured between the 18 and 40 Hz frequency band and Gamma waves are measured between the 40 and 100 Hz frequency band. The Emotiv EPOC+ was previously validated and used to evaluate the immediate effects of manual therapy on the brain [25,26,27,28,29]. The headset was placed on the head following the international 10–20 system for EEG measures [30]. EEG measures allow changes in brain wave activity to be identified and compared to a baseline measure for each wave under each electrode. Event-related brain potentials (ERPs) represent significant changes in brainwave activity and typically meet a 70 mv change threshold [27,28]. However, EEG measures are performed in real time, and thus, there is the potential for physiological artifacts to be generated by the subjects, which could create ERPs that lead to measurement error. To minimize such an effect, the brainwave measurement protocol was time-based (15 s measures) regardless of whether they resulted in an ERP (Table 1). This methodology was used in this study so that both the location of brain changes and the type of wave changes could be observed. The applicant was positioned supine after Researcher I applied the Emotiv EPOC+ and verified connectivity through the Emotive system.

#### 2.2.2. Suboccipital Release

The suboccipital muscle release technique is a method used to achieve relaxation in the suboccipital muscles located between the external occipital protuberance and the upper cervical vertebrae [11]. The technique has been reported to inhibit tone, reduce stress on the surrounding tissues, and have an immediate positive effect, reducing sympathetic nervous system activity [15]. Typically, clinicians place their hands under the individual’s occiput and apply direct pressure to the area between the occiput and axis with their fingers [11,15]. Next, an anterior and cranial directed force is applied through the clinician’s fingers directly into the soft tissues. The applied force is maintained for up to 5 min or until the clinician feels tissue relaxation [15].

#### 2.2.3. Procedure

**Figure 2:**
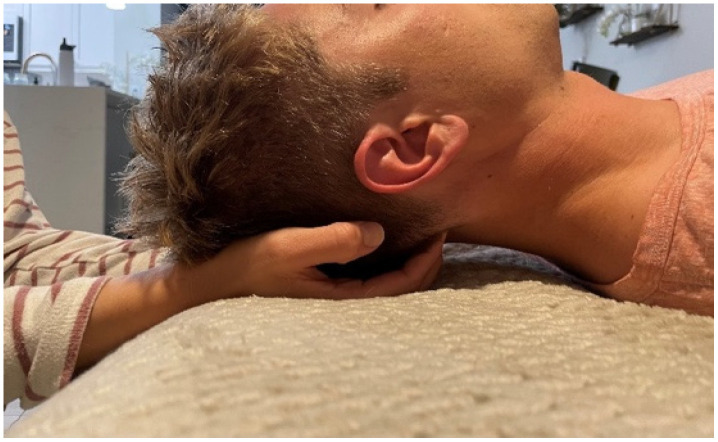
Supine head hold position. The subject’s occipital region rests in the palms of the researcher’s hands. The researcher’s fingertips are directly positioned on the suboccipital muscles and create a posterior to anteriorly directed force that is enough to reach between the occipital bone and the transverse process of axis.

Once the subject was supine with a towel rolled underneath the head, they relaxed their eyes, muscles, and minds. A 15 s EEG was recorded to obtain baseline data (Table 2). Next, the subject’s head was placed in a placebo hold by placing the subject’s occipital area in the palm of both hands of Researcher II (Figure 2) while their eyes remained closed. After this, a second 15 s EEG recording was taken. This measure was used to determine the brain’s response to tactile stimuli prior to the suboccipital release. Following the second EEG measure, Researcher II flexed the metacarpal phalangeal joints while extending the proximal and distal interphalangeal joints in both hands. This places the fingertips directly in the suboccipital space and lifts the head off the towel [11,15]. This position was maintained until Researcher II felt a relaxation of the suboccipital muscles, characterized by a slow posterior rotation of the head toward the examination table. At this point, the third 15 s EEG recording was taken. After their head was released, the subject remained supine with their head on the towel, and their eyes closed while a fourth and final EEG measure of 15 s was recorded.

Following the completion of the EEG measurements, the EPOC+ device was removed from the subject’s head, and then the Global Rating of Change Scale (GROC) was used. The GROC scale is reported to have high reliability and validity with an intraclass correlation coefficient of 0.90.34 [31]. Since no change over time was required, the GROC was an appropriate tool [32]. The GROC quantifies the self-perceived rate of change in muscle tone with answer options ranging from −5 (strongly disagree) to +5 (strongly agree).

## 3. Results

Data were analyzed using IBM^®^ SPSS^®^ Statistics version 28. All data were analyzed using a confidence interval of 95% and a significance level of 0.05. A total of 37 asymptomatic subjects were recruited during a three-month period. All 37 subjects completed the study protocol. Twenty subjects were female (54%) and seventeen were males (46%), with a mean age of 24.5 (SD = 3.67). The brainwaves for each band (Theta, Alpha, Beta, and Gamma waves) were analyzed for normal distribution using the Shapiro–Wilk normality test. Data were not normally distributed (*p* < 0.05); for that reason, the assumption of parametric statistics was not satisfied. Therefore, Friedman’s test determined any significant change between the four measuring time points. If significance was found, the Wilcoxon’s signed rank test was used to determine where this significant change (*p* < 0.05) occurred between the baseline data, physical touch, suboccipital muscle release, and post-intervention relaxation data for each of the five wave types.

**Figure 3:**
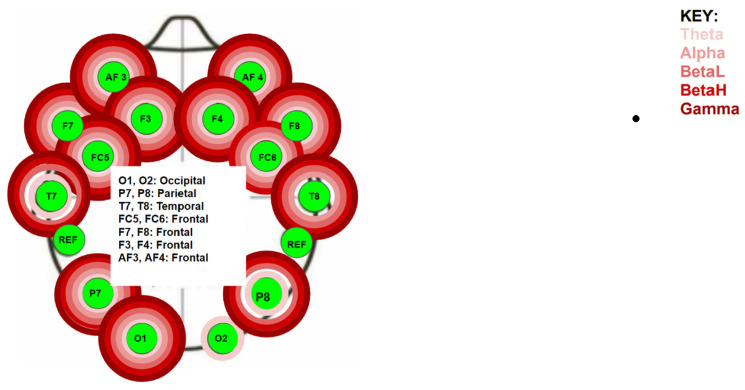
Location of electrodes and waves found to be statistically significant by the Friedman Test.

The EPOC+ measures five different brainwaves (Theta, Alpha, Beta low, Beta high, and Gamma waves) under 14 different electrodes positioned on the skull using the 10/20 method. The Friedman test was used to determine if there was a significant difference for each different brainwave band under each electrode during the experiment. There was a statistically significant difference (*p* < 0.005) at the following electrodes (Figure 3):

- Left frontal lobe: the AF3 electrode for all five bands (*p* < 0.01), the F7 electrode for all five bands (*p* < 0.03), the F3 for all five bands (*p* < 0.35), and the FC5 for all five bands (*p* < 0.01);
- Left temporal lobe: the T7 electrode for the Theta, Beta L, Beta H, and Gamma bands (*p* < 0.02);
- Left parietal lobe: the P7 electrode for all five bands (*p* < 0.04);
- Left occipital lobe: the O1 electrode for all five bands (*p* < 0.01);
- Right occipital lobe: the O2 electrode for the Theta band (*p* < 0.01);
- Right parietal lobe: the P8 electrode for all five bands (*p* < 0.04);
- Right temporal lobe: the T8 electrode for all five bands (*p* < 0.02);
- Right frontal lobe: the FC6 electrode for the Theta, Alpha, Beta L, and Beta H bands (*p* < 0.02), the F4 electrode for all five bands (*p* < 0.01), the F8 electrode for all five bands (*p* < 0.01), and the AF4 electrode for all five bands (*p* < 0.01).

The Wilcoxon test was used to determine where the significant changes occurred between each measuring point under each electrode for all five wavebands. Table 3 displays the recording points and the physical contact between the subject and the researcher. All findings were significant except under the following electrodes (*p* > 0.05):

- Left temporal lobe: the T7 electrode for the Alpha and Beta H bands;
- Right occipital lobe: the O2 electrode for the Beta L, Beta H, and Gamma bands;
- Right parietal lobe: the P8 electrode for the Alpha band;
- Right temporal lobe: the T8 electrode for the Theta band;
- Right frontal lobe: the FC6 electrode for the Gamma band, the F4, F8 and AF4 electrodes for all bands.

**Table 3:** Statistical findings using the Wilcoxon signed-rank test for each waveband and electrode (H = High, L = Low, R1 = head hold position, R2 = suboccipital release, R3 = immediately following release, R4 = final measure). Statistical significant findings are displayed in bold.

F8 Wave	R1–R2	R1–R3	R1–R4	R2–R3	R2–R4	R3–R4
Theta	0.145	** <0.001 **	0.294	** 0.005 **	0.572	** 0.002 **
Alpha	0.592	** <0.001 **	**0.024**	** <0.001 **	0.167	** 0.009 **
Beta L	** 0.033 **	** <0.001 **	0.105	** <0.001 **	0.946	** 0.003 **
Beta H	0.483	** <0.001 **	0.294	** 0.003 **	0.678	** 0.007 **
Gamma	0.780	** 0.002 **	0.769	** 0.008 **	0.839	** <0.001 **
FC5 Wave	R1–R2	R1–R3	R1–R4	R2–R3	R2–R4	R3–R4
Theta	0.084	** 0.012 **	0.294	** 0.001 **	0.455	** 0.023 **
Alpha	0.502	** 0.005 **	0.288	** <0.001 **	** 0.049 **	** 0.049 **
Beta L	0.369	** 0.035 **	0.377	** <0.001 **	0.158	0.053
Beta H	0.678	** 0.029 **	0.645	** <0.001 **	0.420	0.051
Gamma	0.561	0.099	0.635	0.678	** 0.015 **	**<0.001**
FC6 Wave	R1–R2	R1–R3	R1–R4	R2–R3	R2–R4	R3–R4
Theta	0.065	** <0.001 **	0.236	** 0.016 **	0.346	** 0.019 **
Alpha	0.261	** <0.001 **	0.087	** 0.001 **	0.108	** 0.041 **
Beta L	** 0.018 **	** <0.001 **	0.316	** 0.005 **	0.561	** 0.002 **
Beta H	0.158	** <0.001 **	0.492	** 0.017 **	0.394	0.051
Gamma	N/A	N/A	N/A	N/A	N/A	N/A
O1 Wave	R1–R2	R1–R3	R1–R4	R2–R3	R2–R4	R3–R4
Theta	0.521	** 0.041 **	0.862	**0.039**	0.274	** 0.001 **
Alpha	0.187	** 0.010 **	0.757	0.062	0.455	** <0.001 **
Beta L	0.225	** 0.027 **	0.492	** 0.022 **	0.230	** <0.001 **
Beta H	0.502	** 0.020 **	0.386	0.087	0.236	** 0.001 **
Gamma	0.464	** 0.009 **	0.512	0.099	0.281	** <0.001 **
O2 Wave	R1–R2	R1–R3	R1–R4	R2–R3	R2–R4	R3–R4
Theta	** 0.049 **	**0.035**	0.862	0.133	0.242	** 0.006 **
Alpha	0.862	0.354	0.624	0.338	0.338	0.079
Beta L	N/A	N/A	N/A	N/A	N/A	N/A
Beta H	N/A	N/A	N/A	N/A	N/A	N/A
Gamma	N/A	N/A	N/A	N/A	N/A	N/A
P7 Wave	R1–R2	R1–R3	R1–R4	R2–R3	R2–R4	R3–R4
Theta	0.081	** 0.004 **	0.898	** 0.030 **	0.512	** 0.007 **
Alpha	0.108	** 0.002 **	0.323	** 0.005 **	0.958	** 0.027 **
Beta L	0.090	** 0.004 **	0.734	** 0.019 **	0.624	** 0.015 **
Beta H	0.163	** 0.004 **	0.827	** 0.015 **	0.464	** 0.046 **
Gamma	0.242	** 0.006 **	0.958	** 0.013 **	0.678	** 0.012 **
P8 Wave	R1–R2	R1–R3	R1–R4	R2–R3	R2–R4	R3–R4
Theta	0.309	** 0.004 **	0.582	** 0.012 **	0.958	**0.004**
Alpha	N/A	N/A	N/A	N/A	N/A	N/A
Beta L	0.474	** 0.013 **	0.369	** 0.006 **	0.982	** 0.003 **
Beta H	0.346	** 0.005 **	0.242	** 0.006 **	0.734	** 0.004 **
Gamma	0.323	** 0.007 **	0.248	** 0.004 **	0.592	** 0.003 **
T7 Wave	R1–R2	R1–R3	R1–R4	R2–R3	R2–R4	R3–R4
Theta	**0.010**	**0.037**	**0.015**	0.150	0.827	0.464
Alpha	N/A	N/A	N/A	N/A	N/A	N/A
Beta L	0.062	0.613	0.071	** 0.002 **	0.667	** 0.017 **
Beta H	N/A	N/A	N/A	N/A	N/A	N/A
Gamma	0.757	** 0.027 **	0.827	** 0.013 **	0.803	** <0.001 **
T8 Wave	R1–R2	R1–R3	R1–R4	R2–R3	R2–R4	R3–R4
Theta	N/A	N/A	N/A	N/A	N/A	N/A
Alpha	0.122	** 0.009 **	** 0.013 **	0.069	0.108	0.301
Beta L	0.850	0.167	0.093	0.105	0.994	0.994
Beta H	0.723	0.172	0.361	0.038	0.316	0.592
Gamma	0.323	** 0.007 **	0.248	** 0.004 **	0.592	** 0.003 **

**Figure 4:**
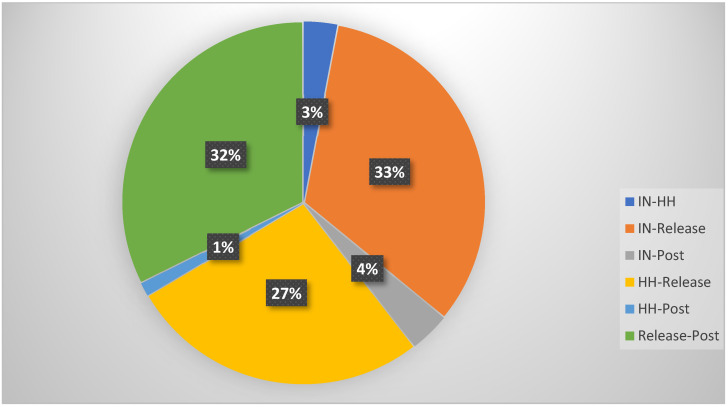
Comparison of significant measurements with Wilcoxon signed-rank test. In = baseline position, HH = head hold position, Release = suboccipital release, Post = resting position after intervention.

The percentage of difference between the four recording points was determined across wave bands for the combined subject pool and is displayed in Figure 4. Most changes in brain wave activity took place between the initial measure and the SRT, between the head hold and the SRT, and following the SRT.

Each subject completed a Global Rating of Change Scale following the EEG recording phase. Using a 5-point Likert scale, four subjects reported feeling unchanged after the measurement phase (10.8%). In total, 80.9% of subjects reported a positive change, as follows: seven subjects reported a “1“ score of change (18.9%), three subjects reported a “1.5” score of change (8%), eight subjects reported a “2” score of change (21.6%), one subject reported a “2.5” score of change (2.7%), seven subjects reported a “3” score of change (18.9%), three subjects reported a “4” score of change (8.1%), and one subject reported a “5” score of change (2.7%). A total of 8.1% of the total subjects reported a negative change following the experiment. One subject reported a “−1” score of change (2.7%), and two subjects reported a “−3” score of change (5.4%). There was an 8-point range in the GROC scores, with a mean score of 1.649 (SD = 1.719 and SE = 0.283), supporting the hypothesis of a self-perceived benefit from the intervention.

## 4. Discussion

This study aimed to determine the immediate effect of the SRT on brainwave activity in a subgroup of healthy individuals. Asymptomatic individuals were chosen for this study as they have similar percentages of trigger points compared to symptomatic individuals [10]. It was hypothesized that reducing muscle tone would change brainwave activity regardless of whether dysfunctions were present. The observed changes in brainwave activity should concurrently relate to their experience.

The upper cervical spine is represented by complex anatomical relationships between tissues such as muscle, ligamentous, capsule, and the dura mater. Abnormal muscular tone is reflective of movement dysfunction and can lead to presentations such as cervicogenic headaches [33]. Forward head posture is the most common positional default, leading to movement dysfunction. Fernández-de-las-Peñas et al. [34] demonstrated that the degree of forward head posture is positively correlated with the presence of suboccipital trigger points. Muscles with trigger points have an altered tone. Previous studies identified that the SRT could result in an immediate change in muscle tone [11,15]. This correlates with the fact that the subjects in this study experienced a positive change. This also concurs with the findings of Jiang et al. [35], who demonstrated that the use of SRT was beneficial in the treatment of the subjects’ headaches. The clinical significance is that the subjects’ reports support the perception that the SRT results in a positively experienced change.

The systemic effect of the SRT has been postulated previously; however, there is conflicting evidence to support this hypothesis [11,15,16]. Sillevis et al. [11] could not identify immediate significant autonomic nervous activity following the SRT, while Metzler-Wilson et al. [16] demonstrated the immediate effect of the SRT on the autonomic nervous system. The results of this study support the hypothesis that the SRT has a systemic effect, as significant changes in brainwave activity were identified (*p* < 0.05) under most EEG electrodes over various brainwave bands.

This study did not control for the possible factors contributing to the physical touch response. One’s response to physical touch is affected by various factors such as the tissues being touched, the way the touching takes place, emotions, genetics, current thoughts, previous experiences, and the subject’s psychology [36,37]. The relationship between neurophysiology and sensation perception indicates a need to comprehend how the brain perceives sensory stimuli. The results of the current study provide a brain map response to the SRT. Although the existing research on tactile stimuli and brain waves is limited, there are studies related to this topic [37,38]. The response of brain wave activity to touch appears independent of visual sensory input; it are dependent on how pleasant the touch stimulation is experienced [37,38,39]. The nervous system can process touch independently in the brain despite input from other systems [30]. Singh et al. [38] demonstrated that pleasant touch suppresses Alpha and Beta band activity in electrodes opposite the side of the touch. This relationship was conversely correlated with the intensity of the individual’s experience.

Touch is processed in the brain in the parietal (EPOC+ P7 and P8 electrodes) lobe and the somatosensory cortex (EPOC+ FC5 and FC6 electrodes). In our subject sample, the Alpha and Beta wavebands under the P7, FC5, and FC6 electrodes significantly decreased (*p* < 0.05). Additionally, the beta band under the P8 electrode decreased significantly (*p* < 0.05). This implies that our subject sample experienced the SRT intervention positively, and that it created overall relaxation. This finding correlates well with the reported self-perception of most subjects in this study. Most subjects (80.9%) reported an overall positive change following the SRT. This percentage of change correlates to the 81.5% reported by Sillevis [11] in a previous study on the effects of the SRT. Although this study had a subgroup of asymptomatic subjects, Rodriguez-Huguet et al. [12] reported a similar observation in symptomatic subjects.

Both the head hold and the SRT intervention resulted in a direct change in brainwave activity, thus implying a direct response to physical touch. Since the subject’s position on the table did not change, sensory input could not account for any observed EEG changes. When comparing the subject sample as a whole, only 3% had a significant change (*p* < 0.05) from the initial EEG recording (overall band waves) to the head hold position. The SRT resulted in a 33% significant change (*p* < 0.05) in the overall band waves recorded by the EEG for this subject sample. This implies that the observed EEG changes reflect the actual systemic effect of the SRT.

Previous experience with the SRT or simply an expectation bias could result in added effects beyond those of the intervention. Therefore, any therapeutic intervention could result in a placebo-like response. Gamma waves are directly related to perception [26]. If the placebo effect contributed to the changes observed in this study, it would be expected that the Gamma brainwave activity in the frontal lobe would decrease [40,41]. The EEG changes in this study demonstrated an increase in frontal lobe activity, making it less likely that the observed brainwave changes following the SRT resulted from a placebo effect. Despite this finding, it cannot be ruled out that there were possible placebo effects, which could have caused the changes in the EEG activity observed in this study. This possible placebo effect will be challenging to control in future studies.

### Limitations

The design of this study has several limitations. First, it had a small subject sample, limiting the findings’ generalizability. Additionally, gender representation was unequal, as the subject sample included 12 males and 15 females. However, this is unlikely to have negatively affected this study’s outcome since it was previously demonstrated that gender differences do not impact systemic responses to stimuli [42]. Although only asymptomatic individuals participated in this study, it is possible that the initial application of the force through the fingers of the clinician to the suboccipital region was uncomfortable for the subjects and thus affected the EEG measurements. The asymptomatic subjects were selected through convenience sampling. The results could be different in subjects with painful conditions elsewhere in the body, thus affecting EEG activity, a consideration for follow-up studies. Since pain directly stimulates the central nervous system, follow-up studies should evaluate if a “pain subject group” would respond differently to the SRT than the subjects in this pilot study. Lastly, the subjects in this study served as their own control.

## 5. Conclusions

The results of this study indicate that the suboccipital release technique has a significant immediate effect on brain wave activity throughout different regions of the brain. This change is likely the result of a systemic response and not the result of any placebo effect, and correlates highly with the subject’s self-perception of a change following the intervention. The results of this study offer EEG brainwave mapping following a suboccipital muscle release in healthy asymptomatic individuals. Overall, the finding supports the clinical use of the SRT if a centralized effect on the nervous system is desired. Future studies should evaluate the SRT’s effect on symptomatic individuals based on the established EEG brain map following the SRT.

## Figures and Tables

**Table 1:** Timeline of electroencephalogram (EEG) measurements.

	Participant Relaxation (Initial)	Placebo Head Hold (HH)	Suboccipital Release (Release)	Participant Relaxation (Post)
Time of each measurement (s)	15 s	15 s	15 s	15 s
Total time of EEG recording (s)	15 s	30 s	45 s	60 s

**Table 2:** Measure point with position and contact.

Recording	Time	Position	Tactile Touch
R1	Immediately before head hold	Supine with head on towel roll position	Absent
R2	Head hold	Supine with head in hands researcher	Present
R3	Suboccipital release	Supine with fingers in suboccipital region and head free	Present
R4	Following release	Supine with head on towel roll position	Absent

## Data Availability

The original contributions presented in the study are included in the article; further inquiries can be directed to the corresponding author.
